# SPOP suppresses tumorigenesis by regulating Hedgehog/Gli2 signaling pathway in gastric cancer

**DOI:** 10.1186/s13046-014-0075-8

**Published:** 2014-09-11

**Authors:** Chunyan Zeng, Yao Wang, Quqin Lu, Jiang Chen, Junyan Zhang, Tao Liu, Nonghua Lv, Shiwen Luo

**Affiliations:** Center for Experimental Medicine, The First Affiliated Hospital of Nanchang University, No. 17 Yongwai Street, Donghu District, Nanchang, Jiangxi 330006 China; Department of Biostatistics & Epidemiology, School of Public Health, Nanchang University, Nanchang, Jiangxi 330006 China; Institute of Digestive Disease, The First Affiliated Hospital of Nanchang University, Nanchang, Jiangxi 330006 China

**Keywords:** SPOP, Gli2, Hedgehog, Degradation, Apoptosis, Gastric cancer

## Abstract

**Background:**

Recent evidence suggests that aberrant activation of Hedgehog (Hh) signaling by Gli transcription factors is characteristic of a variety of aggressive human carcinomas including gastric cancer. Speckle-type POZ protein, SPOP, is an E3 ubiquitin ligase adaptor, and it is found to inhibit oncogenic signaling. However, the molecular mechanisms are largely unknown.

**Methods:**

In this study, we characterized the expression of SPOP in 88 pairs of gastric cancer tissues and adjacent tissues by immunohistochemical staining and Western blotting. The relationship between SPOP expression and clinical pathologic factors was analyzed. Transfected gastric cancer cell lines were used in cell viability, wound healing and colony formation assays. The interaction of SPOP with Gli2 and other related apoptotic proteins was assessed by immunoprecipitation, Western blotting, real-time PCR and dual luciferase reporter assays. Intracellular interaction of SPOP and Gli2 was visualized by immunofluorescent staining in gastric cancer cells.

**Results:**

Immunohistochemical staining of SPOP can be detected in gastric cancer tissues but much less than adjacent gastric tissues (*P* < 0.01). High SPOP expression is negatively correlated with lymph node metastasis, poor histological differentiation, and tumor malignancy according to TNM staging. In vitro experiments revealed that over-expression of SPOP prevented tumor cells from proliferation, migration and colony formation in gastric cancer cell lines. Likewise, repression of SPOP promoted cell viability, migration, proliferation, and attenuated apoptosis. Mechanistic studies revealed that increasing SPOP accelerated Gli2 degradation but regardless of Gli2 synthesis. Furthermore, cytoplasmic Gli2 decreased markedly along with the abundant expression of SPOP in MKN45 cells.

**Conclusions:**

Our findings indicate that SPOP plays critical roles in suppressing gastric tumorigenesis through inhibiting Hh/Gli2 signaling pathway. It may provide an alternative strategy for developing therapeutic agents of gastric cancer in future.

## Background

Gastric carcinoma (GC), with an estimated number of one million new cases every year, is the fourth most common malignant tumor worldwide and the second most common cause of cancer-related deaths [1,2]. Although extensive research on the molecular mechanisms of GC progression has been carried out, the prognosis of patients with GC is still poor. Therefore, further understanding of the molecular mechanisms of GC progression and the development of new therapeutic targets based on these mechanisms are anticipated.

The Hedgehog (Hh) signaling pathway is crucial for the growth and patterning of various tissues during embryonic development [3]. Aberrant Hh pathway activation, driven by either mutation or ligand overexpression, is often associated with human cancers [4,5]. This pathway is a highly coordinated network of Hh (Sonic Hh, Indian Hh and Desert Hh) proteins, seven-transmembrane protein Smoothened (Smo), 12-transmembrane receptors (Patched1 [Ptch1] and Patched2 [Ptch2]), and transcription factor Gli family (Gli1, Gli2, Gli3) [6-8]. Hh exerts its biological function through a signaling cascade that culminates in an alteration of the balance between activator and repressor forms of the Gli transcription factors. The vertebrate Gli repressor function largely depends on Gli3, while the primary Gli activator function largely depends on Gli2. Gli1 acts as a transcriptional activator and thus a reliable indicator of Hh pathway activity [9,10]. In Drosophila, the Hh signal is mediated by the Cubitus Interruptus (Ci) transcription factor. Yet the Ci function is positively regulated by Fused (Fu) serine/threonine kinase, but suppressed by Suppressor of Fused (SuFu) and the kinesin-like molecule Costal2 (Cos2) [11]. SuFu is antagonized by HIB (Hh-induced MATH and BTB domain protein), of which speckle-type POZ protein, SPOP, is the vertebrate homolog [12].

SPOP is demonstrated as an E3 ubiquitin ligase adaptor. It has 374 residues and contains three domains: an N-terminal MATH domain (residues 28–166) that recruits substrates, an internal BTB domain (residues 190–297) that binds Cul3, and a C-terminal nuclear localization sequence (residues 365–374) [13]. SPOP is mostly studied in ubiquitin-dependent proteolysis, however, its role in tumorigenesis is largely unknown [13,14]. Li C., et al. demonstrated that SPOP is responsible for full length Gli2 and Gli3 ubiquitination and proteolysis [15]. Recently, Geng C., et al. revealed that wild type SPOP functions as a critical tumor suppressor in prostate cancer cells by promoting the turnover of SRC-3 (p160 steroid receptor coactivator-3) protein and, thus suppressing androgen receptor transcription activity [16]. Wang C., et al. showed that SPOP promotes full-length Gli2 and Gli3 degradation, which may indicate a potential tumor suppressor role of SPOP [17]. The limited studies indicate the importance of SPOP in tumorigenesis, which prompts us to find out more evidence of it.

In this study, we evaluated SPOP expression in gastric cancer tissues and adjacent gastric tissues. We then exhibited possible correlations between SPOP expression and the clinical pathologic factors. Based on the results of clinical findings, we performed in vitro experiments and studied the effects of up-regulated or down-regulated SPOP expression on the proliferation, migration and apoptosis of GC cell lines. Our results indicate that SPOP reduces gastric cancer cell invasion and proliferation by regulating Hh/Gli2 signaling pathway.

## Methods

### Constructs, antibodies and reagents

Human SPOP cDNA was amplified by PCR and then inserted into KpnI and NotI sites of Pub6/V5-HisB vector. SPOP - miRNA (miR-SPOP) expressed vectors were generated using the BLOCK-iT™ Pol II miR RNAi Expression Vector Kit (K4936-00, Invitrogen, Carlsbad, CA). Clones were confirmed by DNA sequencing of both strands (Generay, Shanghai, China). The oligonucleotide sequences for miRNA constructs were as follows: for miR-SPOP-607, 5′-AAT GAC TTC ACC CAT TTC CTC-3′; for miR-SPOP-917, 5′-TAA GCT TGT CAT CAG GGA GAA-3′; and for miR-SPOP-1430, 5′-AGT TGA TGA AAT CCA CTG CCT-3′. The numbers in the miRNA constructs indicate the start of targeted nucleotide sequence of the gene. All transfectants were selected with 7 μg/ml Blasticidin for 3 weeks and selected clones were identified by Western blotting. Antibodies were purchased from Abcam (Gli2, ab26056; p16, ab108349; PTEN, ab31392), Cell Signaling (Gli1, L42B10; Caspase-3, 9665S; cleaved Caspase-3, 9664; Ki-67, 9449; Cyclin B1, 4135S; p21, 2947; p27, 3686; Daxx, 4533; p-ERK, 4376S), Novus (SPOP, H00008405-B01P), Protein-Tech (SPOP, 16750-1-AP), Santa Cruz (PCNA, sc-9857; PARP, sc-7150; Actin, sc-1616), Abgent (DUSP7, AP8450a), Millipore (GAPDH, MAB374), Thermo (goat anti-rabbit IgG, 31460). Enhanced chemiluminescence (ECL) Western blot detection reagents were from Thermo Fisher Scientific (Rockford, IL). MG-132 (Z-Leu-Leu-Leu-CHO, I-130) was from BostonBiochem. Protease inhibitor cocktail, Lubrol-PX, and 3-(4,5-dimethylthiazol-2-yl)-2,5-diphenyltetrazolium bromide (MTT) were purchased from Sigma-Aldrich (St. Louis, MO).

### Cell culture and tissue specimens

Human gastric epithelial cell line GES-1, human gastric cancer cell lines AGS, MKN28, SGC-7901, MKN45 and human embryonic kidney 293 T (HEK293T) cell lines were obtained from American Type Culture Collection (ATCC, Manassas). Cells were maintained in DMEM (Invitrogen, CA) supplemented with 10% fetal bovine serum (FBS; Invitrogen, CA) and 100 μg/ml streptomycin in humidified incubator at 37°C with 5% CO_2._

Gastric cancer samples were obtained from 88 patients diagnosed gastric carcinoma from March 2007 to April 2010 in the Department of Pathology, the First Affiliated Hospital of Nanchang University. All the patients had not received chemotherapy or radiotherapy before surgery and diagnosed by clinic pathologic staging based on the 2010 American Joint Committee on Cancer (AJCC) criteria. Detailed clinic pathologic information of all patients and their correlation with SPOP expression were summarized in Table 1. This study was approved by the institutional review board of the First Affiliated Hospital of Nanchang University. All patients had given written informed consent.

**Table 1:** **Correlation between SPOP expression and clinic pathological information**

**Variable**	**Groups**	**SPOP expression scoring**		**Spearman’s** ***R***	***P***
		**Low (<6)**	**High (≥6)**		
Gender	Male	54	10		
	Female	22	2	−0.095	0.380
Age	≤50	18	1		
	>50	62	7	0.070	0.518
Lymp node	Metastasis	54	4		
	No metastasis	23	7	0.236	0.027*
Histopathologic grading	Poorly differentiated	34	0		
	Moderately differentiated	36	10		
	Well differentiated	8	0	−0.232	0.029*
TNM staging	I	13	1		
	II	33	7		
	III + IV	30	4	−0.375	0.0003*

*Significant difference.

### Immunohistochemical staining

Paraffin sections (5 μm) of formalin-fixed gastric cancer tissue and adjacent tissue arrays were de-waxed, rehydrated and incubated in 3% H_2_O_2_ for 10 min to block endogenous peroxidase. Then the sections were autoclaved in 10 mM sodium citrate buffer (pH 6.0) for 15 min. Normal goat serum (10%) was used to block non-specific staining and then the tissue sections were exposed to indicated antibodies. All of the stainings were observed by at least 2 independent investigators blinded to the histopathologic features and patient data of the samples [18]. The widely accepted German semi-quantitative scoring system was used for assessing the staining intensity and area extent [19]. Each specimen was assigned a score according to the intensity of the nucleic, cytoplasmic, and/or membrane staining (no staining, not detected = 0; weak staining, light yellow = 1; moderate staining, yellowish brown = 2; strong staining, brown =3) and the extent of stained cells (no staining = 0, 1-24% = 1, 25-49% = 2, 50-74% = 3, 75-100% = 4). The final immunoreactive score was determined by multiplying the intensity score with the extent score of stained cells, ranging from 0 (the minimum) to 12 (the maximum).

### Western blotting

Cells were solubilized in the extraction buffer (0.5% Lubrol-PX, 50 mM KCl, 2 mM CaCl2, 20% glycerol, 50 mM Tris–HCl, and inhibitors of proteases and phosphatases, pH 7.4) at 4°C for 30 min and centrifuged (12,000 rpm, 15 min at 4°C) to maintain the supernatant. Protein concentrations were determined by Bradford assay (Bio-Rad, CA). Proteins were resolved by SDS-PAGE and transferred to a polyvinylidene difluoride membrane (Bio-Rad), and then probed with the specified primary antibody followed by the appropriate secondary antibody. The immunostaining was visualized by using Kodak X-ray films, and quantified by scanning films containing nonsaturated signals with an Epson 1680 scanner before analyzed with ImageJ software [20].

### Immunoprecipitation

Supernatant of the cell lysates was incubated with indicated antibodies and protein-G agarose beads at 4°C overnight. The beads were washed with the buffer (0.1% Lubrol-PX, 50 mM KCl, 2 mM CaCl2, 20% glycerol, 50 mM Tris–HCl, and inhibitors of proteases and phosphatases, pH 7.4), then subjected to SDS-PAGE and subsequent immunoblotting with antibodies.

### Cell viability analysis

Cell viability assay was performed using 3-(4,5-dimethylthiazol-2-yl)-2,5- diphenyltetrazolium bromide (MTT, Sigma) reduction assay [21]. Briefly, cells were seeded at a density of 300 cells per well in a 96-well plate and cultured for indicated days. Culture medium was replaced with fresh media every day. MTT (20 μl) was added for 4 h to allow formation of the colored formazan product. Then, the supernatant was replaced with 150 μl dimethyl sulfoxide (DMSO). Plates were incubated for a further 2 h and then optical density was measured using a microplate ELISA reader (Bio-Rad) at 490 nm. The relative survival rates were presented as the percentage of control. Triplicate wells were used for each cell line. Each experiment was repeated three times.

### Scratching assay

Cell migration was measured using a scratch assay [22]. AGS cells were plated in 6-well plates to create a confluent monolayer. At a confluence of 90-100%, a 200 μl pipette tip was used to make a “scratch” in the cell monolayer. Then the cells were transfected with SPOP plasmid or empty plasmid as control for 24 h. After removing debris and adding fresh media containing 2% FBS, cells were photographed using phase contrast microscopy (Olympus, IX71). The migration distance was assessed using ImageJ software. A relative migration rate was calculated by cell relative migration rate for each treatment.

### Colony formation assay

One or two hundred cells were plated into 6 cm dishes and incubated in DMEM with 10% FBS at 37°C. Two weeks later, the cells were fixed and stained with 0.1% crystal violet. The number of colonies, defined as >50 cells/colony, were counted. The experiments were performed in triplicate.

### Real-time PCR

Total RNA (1 μg) was employed to prepare cDNA via reverse transcription using cDNA synthesis kit (Invitrogen) according to manufacturer’s instructions and analyzed using an Applied Biosystems 7500 PCR Detection System (Applied Biosystems Inc.). Target gene mRNA expressions were normalized to the expression of GAPDH and quantified using the comparative Ct method. The primers used for real-time PCR are as follows: SPOP (forward, 5′-CAA GGC AAA GAC TGG G-3′; Reverse, 5′-AAC ACT CAC CTC GCA GA-3′); Gli1 (forward, 5′-TCC TAC CAG AGT CCC AAG TT-3′; reverse, 5′- CCC TAT GTG AAG CCC TAT TT-3′); Gli2 (forward, 5′-CCT GGC ATG ACT ACC ACT ATG AG-3′; reverse, 5′-GGC TTG GCT GGC ATG TTG-3′); β-actin (forward, 5′-CGG GAA ATC GTG CGT GAC-3′; reverse, 5′-ATG CCC AGG AAG GAA GGC T-3′).

### Dual luciferase reporter assay

We inserted Gli binding sites (8 × GBS) into plasmid PGL4.2 in HEK293T cells and screened for stable cell lines [23]. Myc-SPOP or empty vector were co-transfected into the cells for 48 h. Luciferase activity was assayed using dual luciferase reporter assay system with procedure described by the manufacturer (Promega, Madison, WI). Change of luciferase activity in treated cells was expressed as percentage of the controls.

### Immunofluorescence analysis

MKN45 cells were transfected with Myc-SPOP, miR-SPOP or empty vector as control. Approximately 48 h after transfection, cells were fixed with 4% paraformaldehyde/phosphate-buffered saline (PBS), permeated with 0.1% Triton/PBS, blocked in 3% BSA/PBS for 30 min, and then incubated with indicated antibodies (1: 200) in 2% BSA/PBS at 4°C overnight. Double-labelled immunostaining was done with appropriate fluorochrome-conjugated secondary antibodies. Protein localization was visualized using a Zeiss-700 (Jena, Germany) confocal microscope.

### Statistical analysis

Values were indicated as mean ± SD. The differences between the groups were evaluated by Student’s *t* test or one-way analysis of variance (ANOVA). For the relationship between SPOP expression and clinical pathologic factors, the Chi-square (*χ*^2^) test, Fisher’s exact test and Spearman’s correlation were used. All analyses were carried out by the use of SPSS17.0 software (SPSS Inc., Chicago, IL). Differences were considered significant if *P* < 0.05.

## Results

### Clinical significance of SPOP expression in gastric cancer

**Figure 1:**
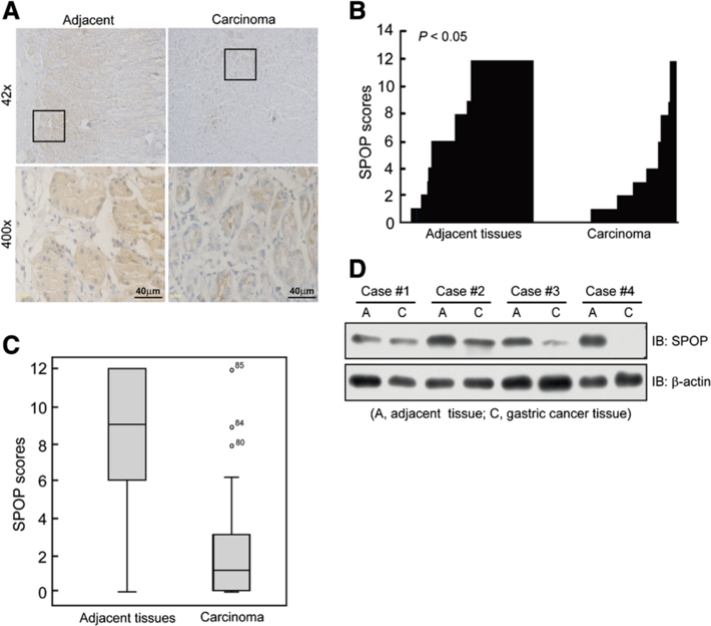
**SPOP expression in gastric cancer. (A)** The representative photos of SPOP expression in gastric cancer tissues and adjacent gastric tissues by using immunohistochemical staining (DAB staining, scale bar, 40 μm). Magnified local images reflecting detailed information were shown on the bottom. **(B, C)** SPOP expression was plotted using the immunochemical scores as described in the Methods. **B**, plot of SPOP scores in each gastric carcinoma and adjacent tissues. **C**, scores of SPOP expression are shown as box plots. The horizontal lines represent the median. The bottom and top edges of the boxes represent the 25th and 75th percentiles, respectively. And the vertical bars represent the range of the data. n = 88. **(D)** Representative immunoblotting of SPOP protein in gastric cancer samples. **C** refers to gastric cancer tissue and **A** refers to paired adjacent non-tumor gastric tissue from the same patient.

Eighty eight gastric cancer cases were eligible for our study. SPOP protein expression was found in most GC tissues and adjacent gastric tissues. Interestingly, the immunostaining of SPOP was much less in GC tissues than in paired adjacent tissues (Figure 1A). In high power field, SPOP was mainly expressed in the cytoplasm and nuclear of adjacent gastric mucosa epithelium cells but less or no staining in gastric cancer cells. The immunohistochemical scoring of SPOP revealed a significant differential staining between GC group and adjacent gastric tissue group (*P* < 0.05), indicating a critical role of SPOP in tumorigenesis of GC (Figure 1B,C). Then, we randomly selected 4 pairs of tissues to detect SPOP protein expression using Western blotting. Consistent with the results above, SPOP protein was found less in GC than in adjacent gastric tissues in most cases (Figure 1D).

We further analyzed the correlation between SPOP expression and clinical pathologic characteristics of patients. Since SPOP scoring ranged from 0 to 12, we defined the score less than 6 as low expression, and equal or more than 6 as high expression. As shown in Table 1, it is notable that SPOP was negatively correlated with lymph node metastasis, poor histopathologic differentiation and advanced TNM stages, regardless of age and gender. Taken together, these results indicate that SPOP is a potential oncogenic suppressor.

### SPOP inhibits gastric cancer cell proliferation and migration

**Figure 2:**
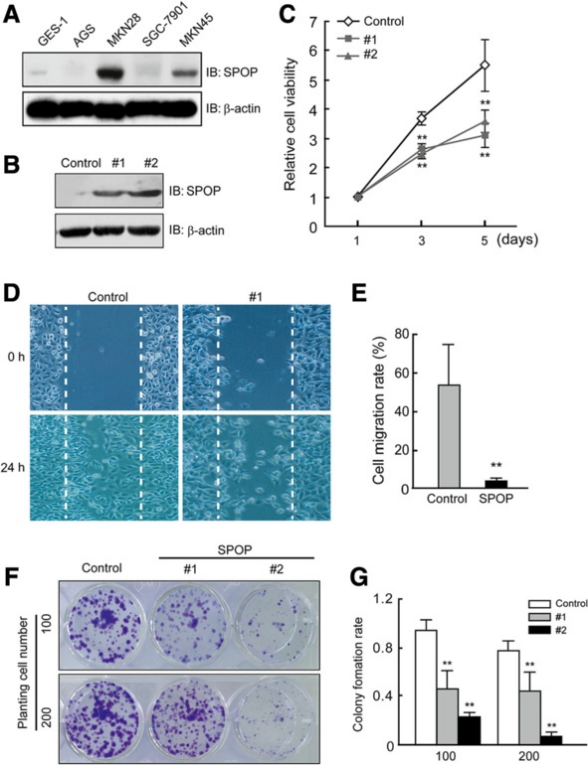
**SPOP inhibits gastric cancer cell proliferation and migration. (A)** The expression of SPOP protein in human GC cell lines - MKN45, MKN28, AGS, SGC7901, and human gastric mucosal cell line GES-1 by Western blotting. **(B)** Detection of SPOP expression in transfected AGS cells. Expression of SPOP was detected in cell lysates of SPOP-V5 transfected AGS cells by Western blotting. Control, AGS cells with empty vector plasmid; #1 and #2 represent different groups of stable AGS cell lines transfected with SPOP. **(C)** Decreased viability of gastric cancer cells transfected with SPOP. The viability of AGS cells was assessed by using an MTT assay. Data represent the average of three experiments (mean ± SD). **P < 0.01, compared to control cells. **(D, E)** Reduced migration of AGS cells following treatment with SPOP transfection. Cell migration was examined using a scratch assay. **D**, images acquired at indicated time points. **E**, quantification of cell migration rates. Data represent the average of three experiments (mean ± SD). **P < 0.01, compared to DMSO treated cells. **(F, G)** Colony formation of SPOP over-expressed AGS cells. One or two hundred stable cell lines with SPOP over-expression were seeded into 12-well plates for 2 weeks. Colonies were fixed and stained with crystal violet, then counted if the colonies > 50 cells. **G**, histograms show the rate of colony formation. Data represent the average of three experiments (mean ± SD). **P < 0.01, compared to DMSO treated cells.

SPOP protein is detected in several human GC cell lines (MKN45, MKN28, AGS, SGC7901) and human gastric mucosal cell line GES-1 by Western blotting assay (Figure 2A). The expression of SPOP protein in different gastric cell lines is imbalanced. We found much higher SPOP expression in MKN28 and MKN45 cells when compared with a relatively low expression in GES-1, AGS and SGC-7901 cells. In order to study effects of SPOP on GC, we generated SPOP transfected AGS cells and two stable cell lines (marked as #1, #2) were screened for further studies (Figure 2B). In MTT cell viability assays, as shown by Figure 2C, cell proliferated in all groups in course of time. As expected, SPOP slowed down proliferation of AGS cells significantly when compared with non-transfected AGS cells (Control). In addition, AGS cell migration was attenuated by over-expressed SPOP in wound healing assay (Figure 2D,E). Colony formation assay revealed that the SPOP transfected AGS cells formed significantly less colonies than control AGS cells (Figure 2F,G). These in vitro results further confirmed that SPOP is a potential tumor suppressor in GC.

### Repression of SPOP promotes proliferation and migration of MKN45 cells

**Figure 3:**
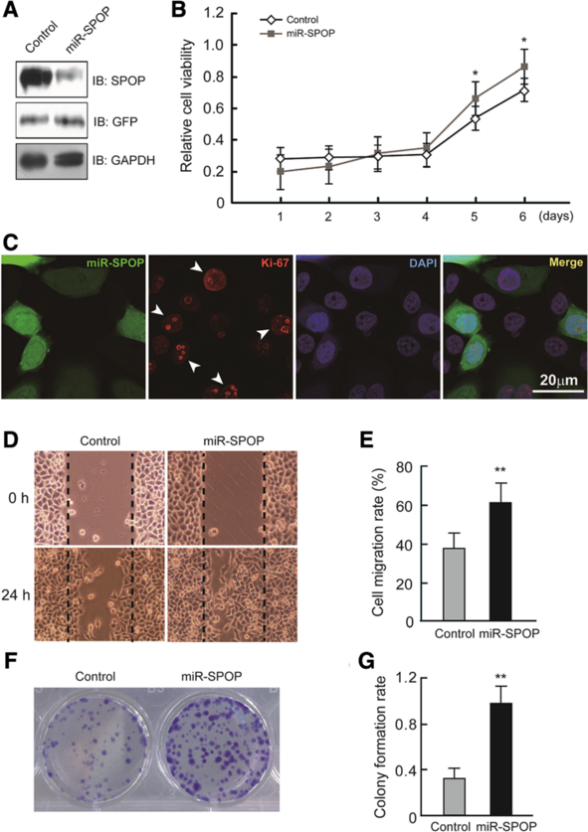
**Repression of SPOP promotes proliferation and migration of gastric cancer cells. (A)** MKN45 cells were transfected with GFP-tagged miR-SPOP and screened by flow cytometry for the stable cells. Western blotting was performed to detect SPOP expression. **(B)** Repression of SPOP enhances gastric cancer cell viability. The viability of MKN45 cells was assessed using an MTT assay. Data represent the average of three experiments (mean ± SD). *P < 0.05, compared to control cells. **(C)** Repression of SPOP promotes Ki-67 expression in nucleus. Immunofluorescent stainings of transfected miR-SPOP and endogenous Ki-67 were performed in MKN45 cells. MKN45 cells were transfected with GFP-tagged miR-SPOP for 48 h. Ki-67 was detected by incubating cells with mouse anti-Ki-67 and subsequently with Alexa Fluor 594 goat anti-mouse antibodies. Nucleus was identified by DAPI staining. **(D, E)** Repression of SPOP accelerates MKN45 cell migration in a scratch assay. Bars represent the standard deviation of three independent experiments. **P < 0.01, compared to DMSO treated control. **(F)** Repression of SPOP increases gastric cancer cell colony formation. Two hundred stable miR-SPOP transfected MKN45 cells were seeded into 12-well plates for 2 weeks. Colonies were fixed and stained with crystal violet and then counted if the colonies > 50 cells. **(G)** Histograms show the colony formation rate. Data represent the average of three experiments (mean ± SD). **P < 0.01, compared to DMSO treated cells.

We next selected MKN45 cells to study the effects of down-regulation of SPOP on cancer cell proliferation and migration. We screened a stable cell line expressing GFP-tagged miR-SPOP in MKN45 cells by flow cytometry after 48 h incubation, and a diminished expression of SPOP protein was detected by Western blotting (Figure 3A). The cell viability of all GFP-tagged cells was suddenly increased from the 5th day of incubation, and the cells with repressed SPOP showed stronger viability than the control (Figure 3B). In order to examine the rate of cell proliferation, we performed immunofluorescent staining of Ki-67 in miR-SPOP transfected MKN45 cells. We observed increased fluorescence intense of Ki-67 in the nucleus of miR-SPOP transfected cells (Figure 3C, indicated with arrow heads). Moreover, repression of SPOP effectively enhanced AGS cell migration and colony formation (Figure 3D-G). Therefore, these results indicate that down-regulation of SPOP expression promotes proliferation and migration of human GC cells.

### SPOP inhibits Hh/Gli2 signaling activity

**Figure 4:**
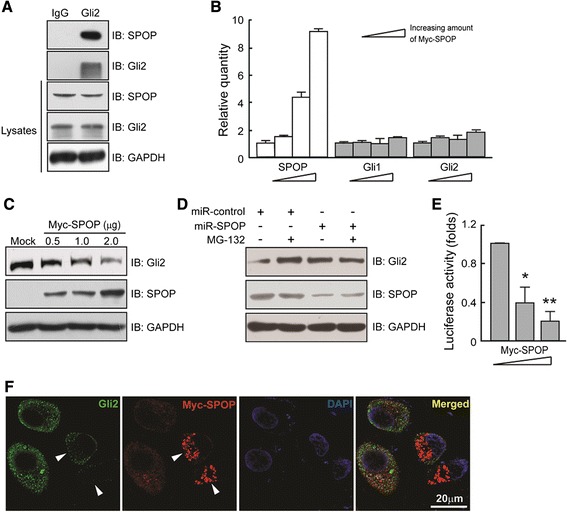
**SPOP inhibits Hh/Gli signaling activity. (A)** SPOP interacts with Gli2 protein in MKN28 cells by co-immunoprecipitation method. Goat anti-rabbit IgG is used as negative control. **(B)** Quantification of Gli1 and Gli2 mRNA in Myc-SPOP transfected MKN28 cells. **(C)** Increasing SPOP reduced Gli2 expression. AGS cells were transfected with different amount of Myc-SPOP for 48 h. Full length of Gli2 is detected in cell lysates by Western blotting. **(D)** SPOP promotes Gli2 degradation through proteasome pathway. MKN45 cells were treated with vehicle (DMSO) or miR-SPOP for 48 h, with or without 10 mM MG-132. In order to limit toxicity, MG-132 was added 4 h before cell harvest. Lysates were subjected to immunoblotting with indicated antibodies. **(E)** Dual luciferase reporter assay is performed in HEK293T cells. Myc-SPOP were transfected into the cells and lysed after 48 h incubation. The percentage of decrease in luciferase activity was calculated. **(F)** SPOP affects Gli2 abundance in cytoplasm. Immunofluorescent stainings of transfected Myc-SPOP and endogenous Gli2 were performed in MKN45 cells. MKN45 cells were transfected with Myc-SPOP for 48 h. Myc-SPOP was detected by incubating cells with mouse anti-Myc antibody and subsequently Alexa Fluor 594 goat anti-mouse antibody. Gli2 was detected by incubating cells with rabbit anti-Gli2 antibody and subsequently Alexa Fluor 488 donkey anti-rabbit antibody. Nucleus was identified by DAPI staining.

To explore the molecular basis that SPOP suppresses tumorigenesis, we studied the relationship of SPOP and Hh signaling pathway which is generally accepted to be involved in tumorigenesis [4,24,25]. Immunoprecipitation showed the interaction of Gli2 with SPOP in MKN28 cells (Figure 4A). With increasing expression of SPOP, mRNA of Gli1 and Gli2 detected by real-time PCR had no significant change (Figure 4B). However, the immunoreactive Gli2 protein decreased in a dose-dependent manner along with the increasing SPOP (Figure 4C). These results indicate a role that SPOP affects Gli2 on post-transcriptional degradation but not on its transcription. A proteasome inhibitor MG-132 was used to inhibit proteasome-dependent degradation of Gli2 protein (Figure 4D). Gli2 degradation was attenuated by treatment with MG-132. However, Gli2 abundance under the treatment of MG-132 can be reduced when SPOP was repressed in transfected cells. These data suggested that SPOP regulates the stability of Gli2 through proteasome-dependent pathway. In addition, dual luciferase reporter assay was performed and we found that increasing Myc-SPOP led to a dose-dependent decrease of Gli transcription activity in HEK293T cells (Figure 4E). We then generated Myc-SPOP transfected MKN45 cells to detect SPOP and endogenous Gli2 expression. We found that the intensity of Gli2 staining was negatively related to SPOP intensity in cytoplasm and perinuclear region (Figure 4F).

### SPOP promotes apoptosis of gastric cancer cells

**Figure 5:**
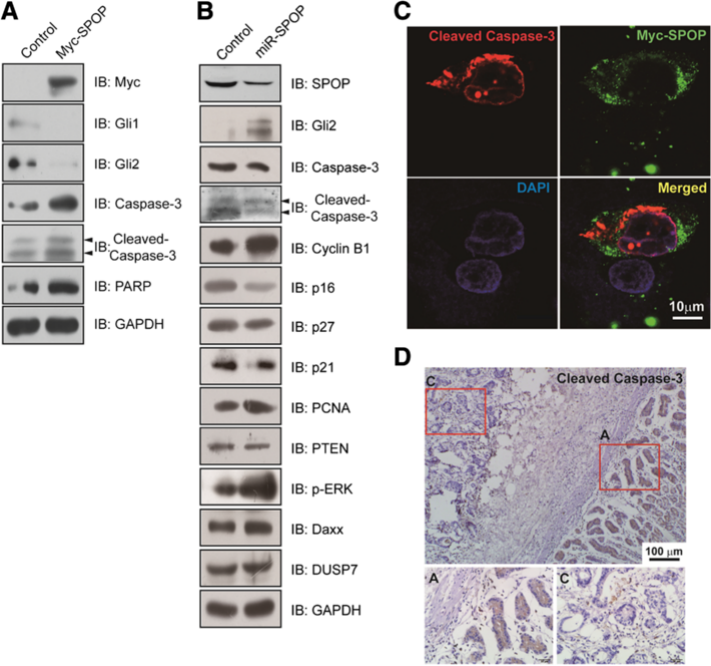
**SPOP promotes tumor cell apoptosis. (A)** Overexpression of SPOP upregulates the expression of apoptotic proteins. MKN45 cells were transfected with Myc-SPOP and incubated for 48 h. Target proteins in cell lysates were detected by indicated antibodies using Western blotting. **(B)** Repression of SPOP inhibits cancer cell apoptosis. MKN45 cells were transfected with miR-SPOP-1430 and incubated for 48 h. Target proteins in cell lysates were probed with indicated antibodies using Western blotting. **(C)** Overexpressed SPOP promotes cleaved Caspase-3 expression. AGS cells were transfected with Myc-SPOP for 48 h and then fixed. Myc-SPOP was detected by incubating cells with mouse anti-Myc antibody and subsequently Alexa Fluor 488 goat anti-mouse antibody. Cleaved Caspase-3 was detected by incubating cells with rabbit anti-cleaved Caspase-3 and subsequently Alexa Fluor 594 donkey anti-rabbit antibody. Nucleus was identified by DAPI staining. **(D)** The representative photos of cleaved Caspase-3 expression in gastric cancer tissues and adjacent gastric tissues by using immunohistochemical staining (DAB staining, scale bar, 100 μm). Magnified local images reflecting detailed information were shown on the bottom.

Several studies indicate that SPOP regulates apoptosis in tumor cells [12,26]. However, apoptotic function of SPOP in gastric cancer was seldom studied. Therefore, we utilize plasmid transfected gastric cancer cells to observe effects of over-expressed or repressed SPOP on apoptosis. As shown in Figure 5A, in Myc-SPOP transfected cells, Hh/Gli pathway related apoptotic proteins, such as Caspase-3, cleaved Caspase-3 and PARP are all increased. Nevertheless, repressed SPOP resulted in decreased Caspase-3, cleaved Caspase-3, tumor suppressor PTEN, and Cyclin-dependent kinase inhibitors p16, p21 and p27 (Figure 5B). In addition, Cyclin B1 which promotes several events of early mitosis, PCNA (proliferating cell nuclear antigen), p-ERK and Daxx are upregulated when SPOP was knockdown. No tendency of DUSP7 abundance was observed (Figure 5B). Since the active form of Caspase-3 (cleaved Caspase-3) marks the frequency of cell apoptosis, we examined the expression of cleaved Caspase-3 in SPOP over-expressed gastric cancer cells and gastric cancer tissues by immunofluorescent and immunohistochemical staining methods. Cleaved Caspase-3 was found intensively stained in Myc-SPOP transfected AGS cells (Figure 5C). In human gastric cancer tissues, immunostaining of cleaved Caspase-3 was mainly found in adjacent gastric tissues but much less in cancer tissues (Figure 5D). Taken together, these results indicate that SPOP plays critical roles in gastric cancer cell apoptosis.

**Figure 6:**
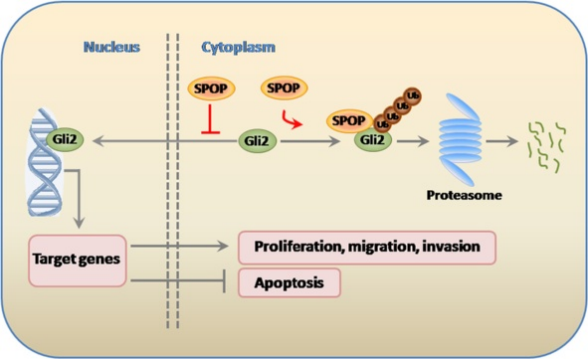
**A working model.** Gli2 is normally kept in the cytoplasm. We propose that SPOP may directly combine with Gli2 and the complex is recognized by ubiquitin. Then the ubiquitinated Gli2 encounters a proteasome-dependent degradation. Or SPOP acts to inhibit Gli2-mediated transcriptional activation and thereby block the effect of Gli2 on the activation of target genes, thus further impact on initiation of cancer cell proliferation, migration, invasion, and apoptosis.

A working model is shown in Figure 6. In brief, Gli2 is normally kept in the cytoplasm. We propose that SPOP may directly combine with Gli2 and the complex is recognized by ubiquitin. Then the ubiquitinated Gli2 encounters a proteasome-dependent degradation. Or SPOP acts to inhibit Gli2-mediated transcriptional activation and thereby block the effect of Gli2 on the activation of target genes, thus further impact on initiation of cancer cell proliferation, migration, invasion and apoptosis.

## Discussion

SPOP is an E3 ubiquitin ligase adaptor and its role in tumorigenesis is largely unknown. In this study, we described the relationship between SPOP expression and clinical pathology parameters of 88 GC cases by immunohistochemical analysis. Interestingly, immunostaining of SPOP is differentially detected in GC tissues and adjacent gastric tissues. Dramatically decreased SPOP expression in GC tissues indicates a role of it in gastric cancer processing. Further correlation analysis revealed that SPOP expression was negatively related to lymph node metastasis, poor histopathologic differentiation and advanced TNM stages. In vitro experiments provide evidence that SPOP functions as a tumor suppressor in different gastric cancer cell lines and contributes to post-transcriptional modification of Gli2. Intracellular studies found that SPOP inhibited Gli2 expression inside the cytoplasm. In addition, SPOP promotes tumor cell apoptosis through regulating the expression of Hh/Gli related apoptotic proteins. Together, our study elucidates the tumor protective function of SPOP through inhibiting Hh/Gli2 pathway, and the possible molecular mechanism of Gli2 stability regulated by SPOP.

Aberrant activation of Hh pathway has been reported in the proliferation of a variety of human cancers, such as BCCs, gastric cancers, prostate cancers, breast cancers, pancreatic cancers [27-34]. As the end effectors of canonical Hh signaling pathway, Gli family play roles of transcription factors, and the abnormal regulation of Gli proteins leads to tumorigenesis [35,36]. Their activity is tightly regulated through various posttranslational modifications, such as ubiquitin-mediated degradation, proteolytic processing, and nuclear-cytoplasmic shuttling. In the present study, Gli mRNA and protein were differentially affected by SPOP in human gastric epidermal cell lines, suggesting a post-transcription modification of Gli by SPOP. As an E3 ubiquitin ligase adaptor, SPOP is supposed to process in degradation of Gli depending on ubiquitin/proteasome system. In fact, a recent study confirmed our speculation [17]. Wang C., et al. found that treatment with proteasome inhibitor MG-132 inhibited SPOP-mediated full-length Gli2 and Gli3 degradation. Co-expression of SPOP and Myc-tagged ubiquitin with either Gli2 or Gli3 resulted in increased levels of ubiquitinated forms of both Gli2 and Gli3 proteins, although the extent of ubiquitination between the two proteins was different. In our study, we demonstrated that increasing exogenous SPOP expression led to decreased endogenous Gli2 both in cytoplasm and nucleus, which provided another direct evidence of Gli2 turnover by SPOP. The biological function of Gli2 is suggested to translocate to the nucleus, where it acts as a transcriptional activator [37]. Actually, we did find endogenous Gli2 expression within the nucleus by immunofluorescent staining, and diminished markedly when SPOP overexpressed. In other words, SPOP interferes Gli2 nucleus/cytoplasm shuttling thus further blocks Gli2 function as a transcription activator in tumorigenesis.

Our proposed degradation of Gli2 by SPOP is analogous to that of Ci by the HIB/Cul3 complex in *Drosophila* [38]. Like HIB, exogenous mammalian SPOP may recruit Cul3 from the cytoplasm along with degradation substrates, likely including Gli2. The molecular basis of Gli2 degradation by SPOP is affected by another inhibitory regulator for Gli proteins-SuFu, which is localized to both the cytoplasm and the nucleus. SuFu sequesters Gli proteins in the cytoplasms, and in the nucleus SuFu plays as a co-repressor of Gli proteins [39]. SuFu and SPOP competitively interact with Gli2 and Gli3 proteins, and SPOP is likely to exhibit a lower binding affinity than SuFu to Gli2 and Gli3 [17]. This might ensure the prompt activation and deactivation of Gli2 and Gli3 proteins in response to Hh signaling.

Limited studies suggest that SPOP also behaves in apoptosis. A study revealed that SPOP BTB protein serves as an adaptor of Daxx, which is a pro-apoptotic protein under various stress condition [12]. Likewise, our data proved that SPOP knockdown by miR-SPOP transfection resulted in reduced expression of Caspase-3, cleaved Caspase-3, p16, p27, and p21 which are cell cycle inhibitors. Furthermore, we found that repressed SPOP promotes early mitosis through enhancing the expression of PCNA and Cyclin B1 respectively. These may indicate a function of SPOP besides E3 ligase adaptor.

Noted that in the control groups of our cultured AGS cell line and MKN45 cell line (Figure 2D,F and Figure 3C,E), under the same incubatory condition, the baseline cell ability of migration and proliferation were different from each other. Lower expression of SPOP may contribute to a more severe malignancy of AGS cells than MKN45 cells.

A recent published study of clear cell renal cell cancer (ccRCC) raises another question that SPOP acts as multiple regulators of cellular proliferation and apoptosis, including not only Gli2 but also tumor suppressor - PTEN, ERK phosphatases and pro-apoptotic molecule Daxx [39]. Thus the total effect of SPOP on clear cell renal cell carcinoma is promoting tumorigenesis. However, in our gastric cancer cell line MKN45, different from ccRCC study, tumor suppressor PTEN was reduced and p-ERK was activated when SPOP was repressed (Figure 5B). These discrepancies indicate multiple roles of SPOP in tumors from different sources of tissues, and the molecular mechanisms are under investigation.

## Conclusions

We report herein that SPOP negatively regulates Hh/Gli2 signaling pathway mediated transcription through interfering Gli2 abundance in gastric cell lines, thus results in decreased tumor cell proliferation, invasion, migration and enhanced cell apoptosis. The identification of SPOP as a negative regulator of Gli2-mediated transcription may provide an alternative strategy for developing therapeutic agents for gastric cancer in future.
